# Implementation and Performance Evaluation of a Bivariate Cut-HDMR Metamodel for Semiconductor Packaging Design Problems with a Large Number of Input Variables

**DOI:** 10.3390/ma14164619

**Published:** 2021-08-17

**Authors:** Yu-Hsiang Yang, Hsiu-Ping Wei, Bongtae Han, Chao Hu

**Affiliations:** 1 Mechanical Engineering Department, University of Maryland, College Park, MD 20742, USA; yhyangv@umd.edu (Y.-H.Y.); hpwei@umd.edu (H.-P.W.); 2 Department of Mechanical Engineering, Iowa State University, Ames, IA 50011, USA; chaohu@iastate.edu

**Keywords:** bivariate cut-HDMR, semiconductor packaging, central composite design, *R*-squared, relative average absolute error

## Abstract

A metamodeling technique based on Bivariate Cut High Dimensional Model Representation (Bivariate Cut HDMR) is implemented for a semiconductor packaging design problem with 10 design variables. Bivariate Cut-HDMR constructs a metamodel by considering only up to second-order interactions. The implementation uses three uniformly distributed sample points (*s* = 3) with quadratic spline interpolation to construct the component functions of Bivariate Cut-HDMR, which can be used to make a direct comparison with a metamodel based on Central Composite Design (CCD). The performance of Bivariate Cut-HDMR is evaluated by two well-known error metrics: *R*-squared and Relative Average Absolute Error (*RAAE*). The results are compared with the performance of CCD. Bivariate Cut HDMR does not compromise the accuracy compared to CCD, although the former uses only one-fifth of sample points (201 sample points) required by the latter (1045 sample points). The sampling schemes and the predictions of cut-planes and boundary-planes are discussed to explain possible reasons for the outstanding performance of Bivariate Cut HDMR.

## 1. Introduction

Numerous metamodeling techniques (also known as response surface methods, surrogate models, or reduced-order models) have been developed and implemented for engineering design optimization [1]. Metamodeling includes two parts: generation of discrete sample points and connection of the discrete sample points. Each metamodeling technique possesses its own characteristics that can be suited for certain applications.

For a typical engineering system, a metamodel considering up to second-order interactions is often sufficient to describe system responses [2,3]. For example, a metamodeling technique called central composite design (CCD) has been implemented widely in the field of semiconductor packaging design community, which uses quadratic polynomial functions for fitting sample points [4,5,6]. It was implemented for commercial software such as optiSLang [7], Design-Expert [8], etc. The CCD metamodeling technique requires P number of sample points to produce the metamodel for N number of input variables, defined as [3]:

(1) $$P=1+2N+2^{N}$$

As the number of input variables increases, the computational cost may become prohibitively high due to an extremely large number of sample points required (this is the well-known “curse of dimensionality”). This situation will be exacerbated when the modeling requires a computationally expensive analysis such as time-dependent and nonlinear analysis that is routinely encountered in complex semiconductor packaging architectures [9,10,11].

In order to build accurate and efficient metamodels for high dimensional input-output systems, numerous advanced metamodeling techniques such as the high-dimensional model representation (HDMR) technique [12,13,14], reduced dimensional polynomial chaos expansion [15], and active and rank-adaptive tensor regression [16] have been developed to enhance the efficiency of metamodeling in various engineering fields.

Among these techniques, one family of HDMR, called Cut-HDMR, possesses two unique practical features: (1) it involves function evaluations only at sample points, and, more importantly, (2) it determines the number of sample points from a pre-defined function of the number of input variables regardless of the nature of engineering applications, i.e., selection of sample points is simple and straightforward [14,17,18]. Based on these features, numerous metamodeling techniques based on Cut-HDMR have been developed such as RBF-HDMR [19], Adaptive MLS-HDMR [20], and Kriging-HDMR [21].

HDMR decomposes a multivariate function into multiple lower-order component functions, based on the hierarchical structure of interaction effects of the input variables. The high performance of some of the metamodeling techniques based on Cut-HDMR considering up to second-order component functions (this will be referred to as Bivariate Cut-HDMR) has been confirmed for nonlinear numerical test functions [19,20] and statistical analysis of multiconductor transmission line networks [22].

The objectives of this paper are (1) to introduce the cut-HDMR to the semiconductor packaging design community and to help implement the Bivariate Cut-HDMR for those who are not familiar with the HDMR, and (2) to investigate the performance of Bivariate Cut-HDMR for a complex semiconductor packaging problem (10 design input valuables). The result is compared with the performance of CCD, which has been utilized widely in the semiconductor packaging industry.

## 2. Background: Bivariate Cut-HDMR

The fundamentals of HDMR are described first. A specific HDMR that uses the Dirac measure located at a cut center, called Cut-HDMR, is presented together with its approximated version, Bivariate Cut-HDMR, which considers up to second-order component functions.

### 2.1. High-Dimensional Model Representation (HDMR)

The concept of high-dimensional expansion was implemented originally to estimate the sensitivity of a function with respect to arbitrary groups of variables [23]. Later, the term, HDMR, was first introduced by Rabitz and Alis [12]. They detailed and completed the general foundations of HDMR.

The HDMR expansion is performed based on the interaction effects of input variables. The term “interaction” employed here means that more than one variable act together to affect the performance function. This is distinctly different from the term “correlation” employed in statistics, which represents whether and how strongly a pair of random variables are related.

A general form of HDMR is defined as [12]:

(2) $$\begin{array}{cc} y\left(x\right) & =y\left(x_{1},\;x_{2},\;\ldots ,\;x_{N}\right)\\ & \equiv y_{0}+\sum_{i=1}^{N}y_{i}\left(x_{i}\right)\;+\sum_{1\leq i<j\leq N}^{}y_{ij}\left(x_{i},\;\;x_{j}\right)+\cdots \;+\sum_{1\leq i_{1}<\cdots <i_{l}\leq N}^{}y_{i_{1}i_{2}\ldots i_{l}}\left(x_{i_{1}},\;\;x_{i_{2}},\;\ldots ,\;\;x_{i_{l}}\right)+\cdots \;+y_{12\ldots N}\left(x_{1},\;\;x_{2},\;\ldots ,\;\;x_{N}\right) \end{array}$$

where y(x) and the bold letter, x, represent the performance function and the vector of input variables, $\left(x_{1},\;x_{2},\;\ldots ,\;x_{N}\right)$, respectively; $y_{0}$ is a constant representing the mean of the performance function, which is called zeroth-order effect or mean effect; $y_{i}\left(x_{i}\right)$ represents the effect when the variable $x_{i}$ acts independently on y(x), which is called first-order effect or main effect; $y_{ij}\left(x_{i},\;x_{j}\right)$ is the effect on y(x) when the variables $x_{i}$ and $x_{j}$ act together, which is called second-order effect or bivariate interaction effect. It should be noted that $y_{ij}\left(x_{i},\;x_{j}\right)$ excludes the main effects of $x_{i}$ and $x_{j}$ as well as the mean effect. The subsequent terms indicate the higher order interaction effects of more variables acting together on y(x). The last term $y_{12\ldots N}\left(x_{1},\;x_{2},\;\ldots ,\;x_{N}\right)$ represents the residual influence.

Each effect in the general form of HDMR is called a component function. The general form of component functions can be expressed as [12]:

(3) $$\begin{array}{ccc} y_{0} & \equiv & My\left(x\right)\\ y_{i}\left(x_{i}\right) & \equiv & M^{i}y\left(x\right)-y_{0}\\ y_{ij}\left(x_{i},x_{j}\right) & \equiv & M^{ij}y\left(x\right)-y_{i}\left(x_{i}\right)-y_{j}\left(x_{j}\right)-y_{0}\\ & \vdots & \\ y_{12\ldots n}\left(x\right) & \equiv & y\left(x\right)-y_{0}-\sum_{i}y_{i}\left(x_{i}\right)-\sum_{ij}y_{ij}\left(x_{i},x_{j}\right)-\ldots -\sum_{12\ldots N}y_{12\ldots N}\left(x_{1},x_{2},\ldots ,x_{N}\right) \end{array}$$

The functions in the above equation are defined as:

(4) $$\begin{array}{c} My\left(x\right)=\int_{K^{n}}y\left(x\right)d\gamma \left(x\right)\\ M^{i_{1}i_{2}\ldots i_{l}}y\left(x\right)=\int_{K^{n-l}}y\left(x\right)\left[\prod_{j\notin \left\{i_{1},\ldots ,i_{l}\right\}}d\gamma_{j}\left(x_{j}\right)\right] \end{array}$$

where $K^{n}=\left\{\left(x_{1},x_{2},\ldots ,x_{n}\right):\;0\leq x_{i}\leq 1,\;i=1,2,\ldots ,n\right\}$ is an *n*-dimensional unit cube and γ is a measure [24]. A measure is a function that quantifies the size of sets. A measure assigns a non-negative real number or +∞ to subsets of a certain set. Each distinct measure embodies a different way to assess how big a set is.

There is no unique decomposition of the model output $y\left(x_{1},\;x_{2},\;\ldots ,\;x_{N}\right)$; all HDMR expansions follow the general form in Equation (2). The choice of a particular HDMR expansion depends on the application and the nature of any constraints in sampling input variables. For example, for the uncertainty analysis of a model output (e.g., an analysis of the variance of an output), the component functions in the HDMR should be chosen to represent the independent contributions of input variables to the overall uncertainty of the output. It is known as ANOVA-HDMR [12,25].

The ANOVA-HDMR is typically carried out by multi-dimensional Monte Carlo integration due to its complexity. The Monte Carlo integration needs a large number of sample points to attain good accuracy. It is impractical for the advanced semiconductor packaging applications where computational cost of each sample point is high. However, another approach of HDMR, Cut-HDMR, can tackle the challenge.

### 2.2. Cut-HDMR and Bivariate Cut-HDMR

Cut-HDMR uses the Dirac measure [26] located at a point $m=\left(m_{1},m_{2},\ldots ,m_{n}\right)$ (also known as cut center):

(5) $$d\gamma \left(x\right)=\prod_{i=1}^{n}\delta \left(x_{i}-m_{i}\right)dx_{i}$$

By combining it with Equations (3) and (4), the component functions of Cut-HDMR can be expressed as:

(6) $$\begin{array}{ccc} y_{0} & \equiv & Mf\left(x\right)=y\left(m\right)\\ y_{i}\left(x_{i}\right) & \equiv & M^{i}y\left(x\right)-y_{0}=y\left(m_{1},\ldots ,m_{i-1},x_{i},m_{i+1},\ldots ,m_{n}\right)-y_{0}\\ y_{ij}\left(x_{i},x_{j}\right) & \equiv & M^{ij}y\left(x\right)-y_{i}\left(x_{i}\right)-y_{j}\left(x_{j}\right)-y_{0}=y\left(m_{1},\ldots ,x_{i},\ldots ,x_{j},\ldots ,m_{n}\right)-y_{i}\left(x_{i}\right)-y_{j}\left(x_{j}\right)-y_{0}\\ & \vdots & \\ y_{12\ldots n}\left(x\right) & \equiv & y\left(x\right)-y_{0}-\sum_{i}y_{i}\left(x_{i}\right)-\sum_{ij}y_{ij}\left(x_{i},x_{j}\right)-\ldots -\sum_{12\ldots N}y_{12\ldots N}\left(x_{1},x_{2},\ldots ,x_{N}\right) \end{array}$$

where $y\left(m_{1},\ldots ,m_{i-1},x_{i},m_{i+1},\ldots ,m_{n}\right)$ is a 1D performance function along the $x_{i}$ direction that passes through m; $y\left(m_{1},\ldots ,x_{i},\ldots ,x_{j},\ldots ,m_{n}\right)$ is a 2D performance function of the $\left(x_{i},\;x_{j}\right)$ plane that passes through m, and so on.

Equation (6) shows that Cut-HDMR is an expression as a superposition of its values on lines, planes, and hyperplanes of higher orders passing through the cut center, m. The expansions of Cut-HDMR do not contain any integral. Cut-HDMR uses only arithmetic computation to determine the component functions, and thus it requires the least amount of computational cost compared to other HDMRs [12,27].

For most well-defined physical systems, the high-order interactions are negligible [14,28], and thus the multivariate performance function of such a physical system can be approximated well by the sum of low-order component functions. Experience shows that an HDMR expansion up to the second-order often provides a satisfactory description of the function for many high-dimensional systems when the input variables are properly chosen [14].

It has been proven that the mean values of input variables, μ, are the optimal cut center m when only the terms up to the second-order are considered [25]. Accordingly, the metamodel based on Bivariate Cut-HDMR can be obtained by substituting Equation (6) into Equation (2) with the cut center being the mean values of input variables. This Bivariate Cut-HDMR metamodel is written as [29]:

(7) $$y\left(x\right)≅\sum_{1\leq i<j\leq N}^{}y\left(x_{i},\;\;x_{j},\;\;\;\mu^{\sim ij}\right)-\left(N-2\right)\sum_{i=1}^{N}y\left(x_{i},\;\;\;\mu^{\sim i}\right)\;+\frac{\left(N-1\right)\left(N-2\right)}{2}y_{0}$$

where $y_{0}=\mu ={\left[\mu_{1},\;\;\;\mu_{2},\;\;\;\ldots ,\;\;\;\mu_{N}\right]}^{T}$ is the vector of the mean values of N input variables (cut center); $\mu^{\sim i}$ is μ without the element $\mu_{i}$; $\mu^{\sim ij}$ is μ without the elements $\mu_{i}$ and $\mu_{j}$; $y\left(x_{i},\;\;\mu^{\sim i}\right)$ is the 1D performance function along the $x_{i}$ direction that passes through μ (cut-line); and $y\left(x_{i},\;x_{j},\;\;\mu^{\sim ij}\right)$ is a 2D performance function on the $\left(x_{i},x_{j}\right)$ plane that passes through μ (cut-plane).

**Figure 1:**
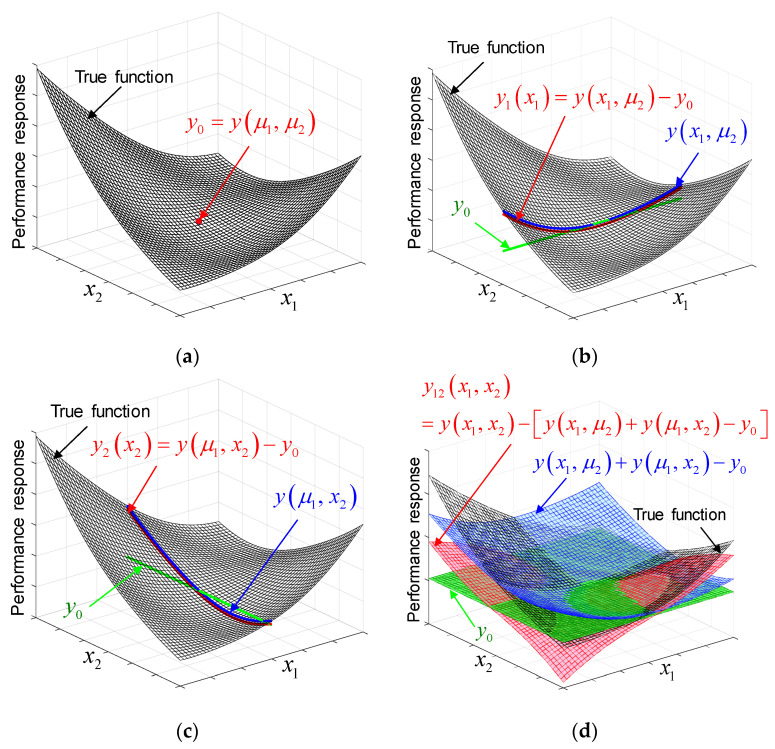
Illustration of Bivariate Cut-HDMR using an arbitrary 2D function; (**a**) 2D function and the effect of zeroth-order, (**b**) the main effect of $x_{1}$, (**c**) the main effect of $x_{2}$, and (**d**) the interaction effect of $x_{1}$ and $x_{2}$.

Figure 1 illustrates the concept of Bivariate Cut-HDMR using an arbitrary 2D function, which is decomposed into four component functions. Figure 1a shows the 2D function, $x^{2}+y^{2}+xy-14x-16y+122=0$, as the black meshed surface, and the dot in the figure represents the zeroth-order effect (i.e., a constant). In Figure 1b, the blue curve is the 1D performance function along the $x_{1}$ direction, in which $x_{2}$ is kept as $\mu_{2}$. The green line is the zeroth-order effect along the $x_{1}$ direction. The main effect of $x_{1}$ is the red curve, obtained by subtracting the green line from the blue curve.

The same procedure can be applied to obtain the main effect of $x_{2}$, as shown in Figure 1c. In Figure 1d, the blue surface is obtained by the superposition of the red curves in Figure 1b,c, which represents the performance function without any interaction effects. The green plane is the zeroth-order effect. By subtracting the blue surface and the green plane from the black surface, the interaction effect of the $\left(x_{1},x_{2}\right)$ pair is obtained, which is shown as the red surface.

## 3. Implementation for Semiconductor Packaging Application

The Bivariate Cut-HDMR technique is implemented to construct a metamodel for a semiconductor packaging application. The application involves warpage prediction of a thin flat ball grid array (TFBGA) package with 10 design input variables.

### 3.1. Description of TFBGA Package

**Figure 2:**
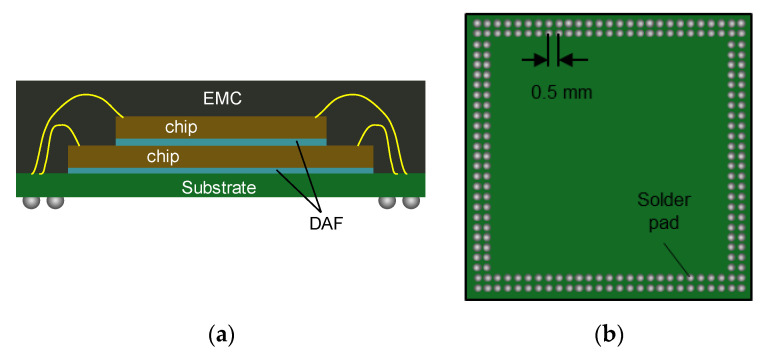
TFBGA package: (**a**) cross-sectional view and (**b**) bottom view.

Figure 2 shows the schematic diagram of a TFBGA package. The first chip is attached to a substrate by the first die attach film (DAF). The second chip is attached to the first chip by the second DAF. Then, they were encapsulated by epoxy molding compound (EMC). A stacked die TFBGA package is often used as the top package of a Package-on-Package (PoP). Warpage at solder pad areas is one of the most critical factors to high PoP stacking yield [30].

**Figure 3:**
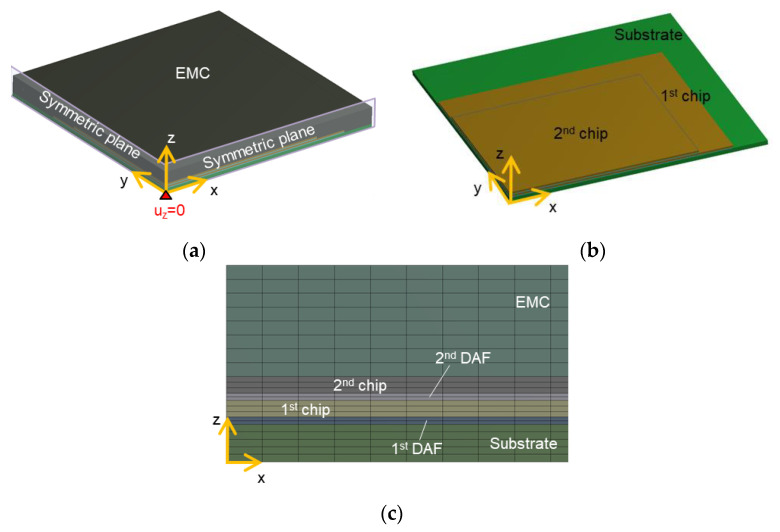
Quarter FE model of TFBGA package: (**a**) boundary conditions; (**b**) die stack configuration; and (**c**) enlarged view of cross-section.

A finite element (FE) model was constructed for warpage prediction. Figure 3 shows details of the FE model built by a commercial FE analysis package (ANSYS^®^). The quarter symmetry model of boundary conditions and the die stack configuration are shown in (a) and (b); and the enlarged view of cross-section is shown in (c). The material properties and the nominal dimensions used in the model are summarized in Table 1 and Table 2. The nominal dimensions of the TFBGA package are adopted from the design in Refs. [10,31].

The TFBGA package was subjected to the EMC molding process at 175 °C, which was used as a stress-free temperature. The conventional lead-free solder reflow profile with the peak temperature of 260 °C was considered [32].

In this implementation, 10 design variables were considered for the warpage prediction of solder pad areas. The details of design variables are summarized in Table 3. The design spaces of the package dimensions and the material properties were defined by the values found in the literature: package dimensions in [31,33,34,35,36,37,38,39] and material properties in [40,41,42,43,44,45].

### 3.2. Sample Points

The number of sample points to construct a Bivariate Cut-HDMR metamodel can be generally expressed as [12]:

(8) $$R=1+N(s-1)+\frac{N(N-1)}{2}{\left(s-1\right)}^{2}$$

where N is the number of input variables and s is the number of sample points taken along the direction of each input variable. N(s−1) points are used to construct 1D performance functions, and $N(N-1){\left(s-1\right)}^{2}/2$ points are used to construct the 2D performance functions.

For the univariate terms (i.e., s number of sample points distributed along each input variable), the center becomes the reference point, and the remaining (s−1) sample points are evenly distributed on two sides with respect to the reference point. For the bivariate terms, the sample points form a uniform gird on a plane with the center as a reference point.

Cut-HDMR, in its original form [14], states that a set of sample points can be selected to calculate the values of corresponding component functions and to form a look-up table that can be used to interpolate component functions at an arbitrary point in the design domain. There has been no universally accepted sampling strategy and interpolation algorithm. The implementation of this study uses three uniformly distributed sample points (s=3) with quadratic spline interpolation to construct the component functions of Bivariate Cut-HDMR. In this way, the Bivariate Cut-HDMR metamodel can be compared directly with the CCD metamodel.

**Figure 4:**
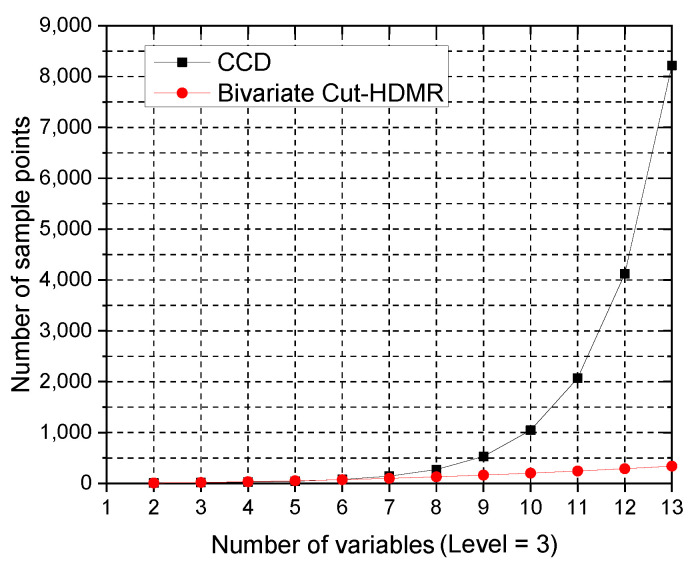
Numbers of sample points required by Bivariate Cut-HDMR and CCD as a function of the number of variables.

Figure 4 and Table 4 show the number of sample points required for the CCD and Bivariate Cut-HDMR metamodels. After N=7, the number of sample points for CCD becomes more than double the number of sample points for Bivariate Cut-HDMR. Considering only the number of sample points, Bivariate Cut-HDMR has a significant advantage over CCD when a metamodeling problem has a large number of input variables.

### 3.3. Construction of Bivariate Cut-HDMR Metamodel

#### 3.3.1. Obtain Sample Points

For *s* = 3, a total of 201 sample points (Equation (8)) are required to construct the Bivariate Cut-HDMR metamodel. The sample points consist of one mean sample point (cut center), 20 univariate sample points, and 180 bivariate sample points.

The mean sample point is the design point, which is the mean values of each design variables. The 20 univariate sample points are the sample points, where one of the design variables takes either maximum or minimum value in its design space while other design variables keep the mean values. The 180 bivariate sample points are the sample points, where two of the design variables take either maximum or minimum value in their design spaces while other design variables keep the mean values. The warpage values of 201 (=1 + 20 + 180) sample points were obtained by the FE model. Since the dimensions of the FE model varies with sample points, the FE model must regenerate different meshes for each sample point. The Supplementary Materials includes: (1) the warpage values of 201 sample points that were used to construct the Bivariate Cut-HDMR metamodel; (2) the warpage values of 1045 sample points that were used to construct the CCD metamodel; and (3) the Monte Carlo simulation sample points used in Section 4.

#### 3.3.2. Construct Performance Functions

After the warpage values at the 201 sample points are obtained, the metamodel can be constructed by applying Equation (7). The quadratic spline interpolation scheme was adopted with all sample points to form the 1D performance functions (cut-lines), $y\left(x_{i},\;\mu^{\sim i}\right)$, and the 2D performance functions (cut-planes), $y\left(x_{i},\;x_{j},\;\mu^{\sim ij}\right)$ by following the procedures below:

- 1D performance functions:
  1. Select a design variable.
  2. Find the three sample points along the design variable that was obtained earlier, i.e., high, mid, and low values of the design variable and other design variables keep the mean values.
  3. Construct the 1D function of the design variable with the three sample points using quadratic spline interpolation. This can be done by using the built-in function that is available in commercial software (e.g., MATLAB).
  4. Select another design variable and repeat steps 2–3 until all 1D performance functions along each design variable are built.

- 2D performance functions:
  1. Select a pair of design variables.
  2. Find the nine sample points along two design variables that were obtained earlier (other design variables keep the mean values) as shown in the figure.
  3. Construct the 2D function of the design variable with the nine sample points using quadratic spline interpolation. This can be done by using the built-in function that is available in commercial software (e.g., MATLAB).
  4. Select another pair of design variables and repeat steps 2–3 until all 2D performance functions of each pair of design variables are built.

**Figure 5:**
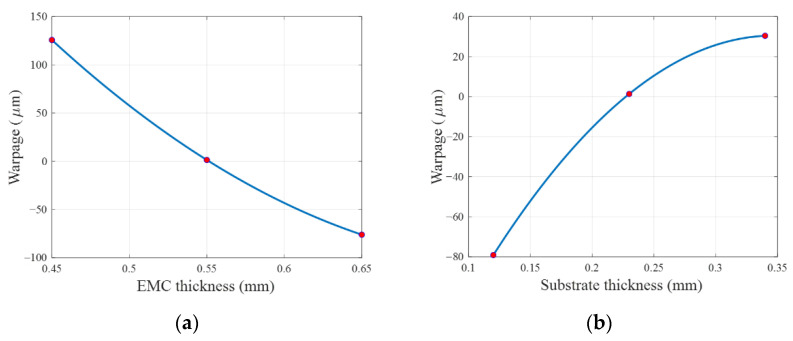
1D performance functions of two design variables: (**a**) EMC thickness and (**b**) substrate thickness.

**Figure 6:**
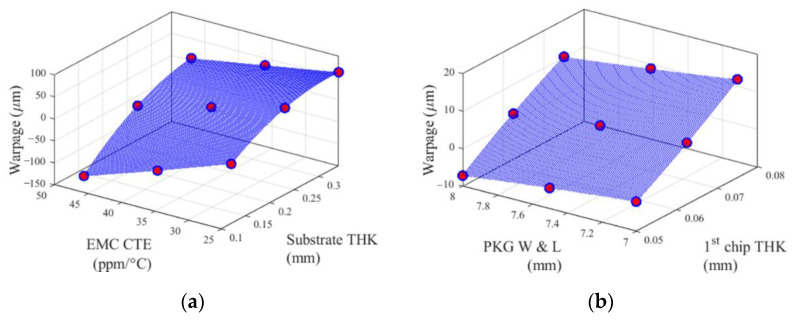
2D performance functions of two pairs of design variables: (**a**) substrate thickness and EMC CTE, and (**b**) package width and length and 1st chip thickness.

The performance functions are illustrated in Figure 5 and Figure 6. Figure 5 shows the 1D performance functions of EMC thickness and substrate thickness, and Figure 6 shows the 2D performance functions of two pairs of design variables. The pair of substrate thickness and EMC CTE has the strongest second-order interaction effect among other pairs. In contrast, the pair of package width and length and 1st chip thickness has the weakest second-order interaction effect. Red dots indicate the sample points used to construct the cut-lines and the cut-planes.

Following is the example of determining the response of a random input by using the constructed metamodel. Assuming that a random input x is (0.81, 0.25, 0.050, 0.064, 0.023, 0.013, 15.3, 10.2, 28.2, 7.69). Equation (7) can be written as:

(9) $$y\left(\begin{array}{l} 0.81,\;0.25,\;0.050,\;0.064,\;0.023,\;\\ 0.013,\;15.3,\;10.2,\;28.2,\;7.69 \end{array}\right)=\sum_{1\leq i<j\leq 10}^{}y\left(x_{i},\;x_{j},\;\mu^{\sim ij}\right)-\left(10-2\right)\sum_{i=1}^{10}y\left(x_{i},\;\mu^{\sim i}\right)\;+\frac{\left(10-1\right)\left(10-2\right)}{2}y_{0}$$

where the warpage at the cut center, $y_{0}=\mu ={\left[\mu_{1},\;\mu_{2},\;\ldots ,\;\mu_{11}\right]}^{T}$, is 0.9 µm; $y\left(x_{i},\;\mu^{\sim i}\right)$ and $y\left(x_{i},\;x_{j},\;\mu^{\sim ij}\right)$ are the values of x on all known 1D performance functions and 2D performance functions that were constructed earlier. Thus, the warpage value at the random input x can be calculated; it was −49.2 µm.

The above Bivariate Cut-HDMR procedure was integrated in MATLAB (R2020b) codes, and they are available at https://www.mathworks.com/matlabcentral/fileexchange/92890-bivariate-cut-hdmr (accessed on 25 May 2021). Those who are interested in implementing Bivariate Cut-HDMR metamodeling can run the script readily by following the instructions.

## 4. Performance Evaluation

The performance of Bivariate Cut-HDMR is evaluated using two well-known error metrics. The performance of CCD is also evaluated for comparison.

### 4.1. Error Metrics

Two error metrics employed to evaluate the performance are: [46]

- Metric 1: *R*-squared

(10) $$R^{2}=1-\frac{\sum_{i=1}^{m}{\left[y\left(x_{i}\right)-\hat{y}\left(x_{i}\right)\right]}^{2}}{{\sum_{i=1}^{m}\left[y\left(x_{i}\right)-\bar{y}\left(x_{i}\right)\right]}^{2}}$$

where m is the number of total test sample points; $y\left(x_{i}\right)$ is a performance function at the *i*th new sample point used for validity check; $\hat{y}\left(x_{i}\right)$ is an approximated performance function at the *i*th new sample point; and $\bar{y}\left(x_{i}\right)$ is the mean of all $y\left(x_{i}\right)$. *R*-squared indicates the overall accuracy of a metamodel, and its maximum value is 1.

- Metric 2: Relative average absolute error (*RAAE*)

(11) $$RAAE=\frac{\frac{1}{m}\sum_{i=1}^{m}\left|y\left(x_{i}\right)-\hat{y}\left(x_{i}\right)\right|}{STD}$$

where *STD* is the standard deviation of all $y\left(x_{i}\right)$. Similar to *R*-squared, *RAAE* quantifies the overall accuracy of a metamodel. The closer a value of *RAAE* is to zero, the more accurate a metamodel is.

Monte Carlo simulation (MCS) was performed to produce 1000 additional sample points. They were used to evaluate the performance of Bivariate Cut-HDMR using the above metrics. The results of the performance metrics are summarized in Table 5. The values of *R*-squared and *RAAE* are 0.9855 and 0.0880, respectively.

A metamodel based on CCD was also constructed for comparison. A total of 1045 sample points were required for the CCD metamodel, which built a 10D quadratic function to define the warpage behavior. The additional sample points obtained from MCS were utilized again to evaluate the performance of CCD metamodel. The results are also shown in Table 5. The values of *R*-squared and *RAAE* are 0.9662 and 0.1472, respectively.

**Figure 7:**
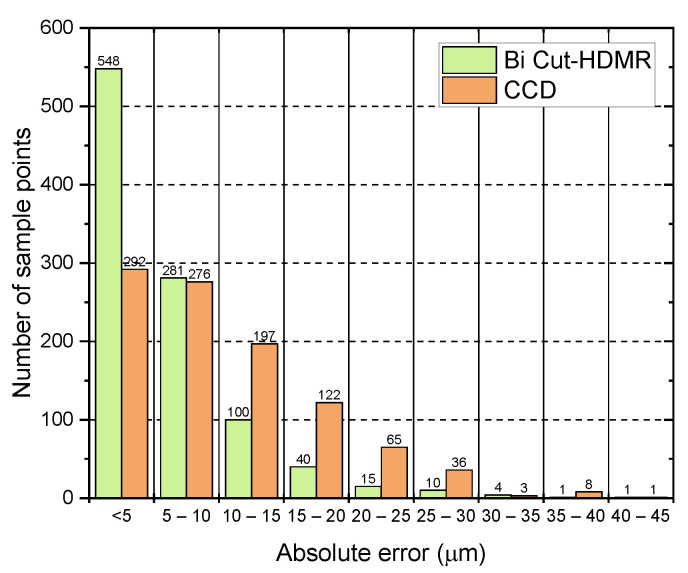
Absolute error of Bivariate Cut-HDMR and CCD of 1000 MCS.

More direct and quantitative comparisons are shown in Figure 7, where the absolute errors of the MCS sample points are compared. The absolute errors of half the MCS sample points of Bivariate Cut-HDMR are less than 5 µm. The outcome is remarkable. Bivariate Cut HDMR used only one-fifth of sample points (201 sample points) required by CCD (1045 sample points). However, Bivariate Cut-HDMR does not compromise the accuracy when compared to CCD. The following section is intended to provide some insight into this performance of Bivariate Cut HDMR.

### 4.2. Discussion: Bivariate Cut-HDMR vs. CCD Metamodel

#### 4.2.1. Sampling Scheme

**Figure 8:**
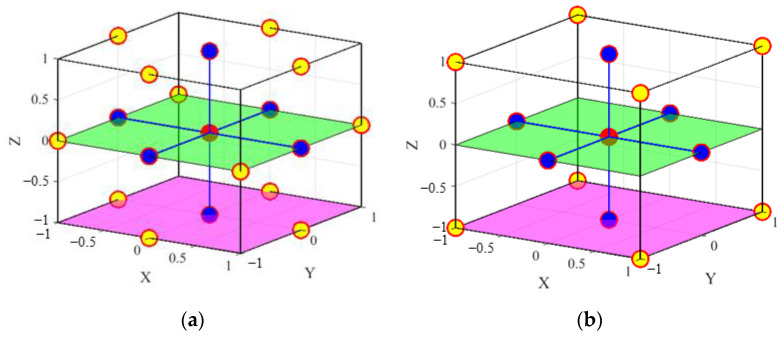
Illustration of sample points for N=3: (**a**) Bivariate Cut-HDMR with s=3 and (**b**) CCD, where the red point is the mean point.

Figure 8 shows the sampling schemes of Bivariate Cut-HDMR and CCD for a three-variable (N=3, s=3) example. In the figure, the red point is the mean point for both Bivariate Cut-HDMR and CCD; the blue points are used to construct the functions of three lines in the X-, Y- and Z-directions for Bivariate Cut-HDMR and the axial points for CCD; and the yellow points together with the blue points are used to construct the functions of three planes (X-Z plane, Y-Z plane, and X-Y plane) for Bivariate Cut-HDMR and the factorial points for CCD. It also illustrates one of the cut-planes (green planes) and one of the boundary-planes (magenta planes) of both metamodels.

The sampling points of Bivariate Cut-HDMR are utilized to construct the first-order and second-order component functions, i.e., every sample point is used to construct the 1D and 2D performance functions (as illustrated in Figure 5 and Figure 6). On the other hand, the sample points of CCD are aimed to cover the boundaries of a design domain.

#### 4.2.2. Prediction of Cut-Planes

As mentioned earlier, the sampling scheme of Bivariate Cut-HDMR is designed to construct the cut-lines and cut-planes. The prediction on the cut-planes performed by both metamodels are compared. As shown in Figure 8, Bivariate Cut-HDMR has more sample points (9) than CCD (5) on the cut-planes (green planes).

**Figure 9:**
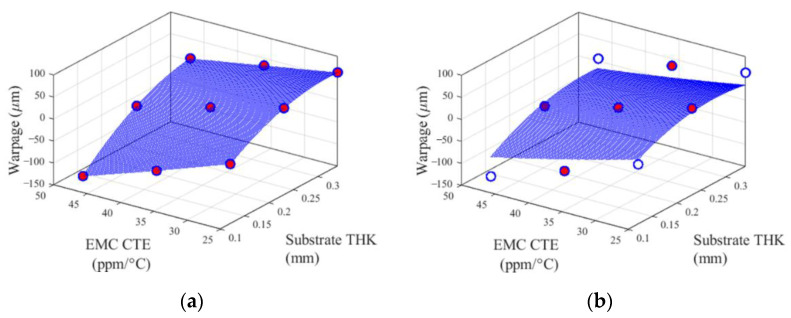
2D performance functions of design variables of (**a**) Bivariate Cut-HDMR and (**b**) CCD, where design variables other than EMC CTE and substrate thickness are kept at their mean values.

**Figure 10:**
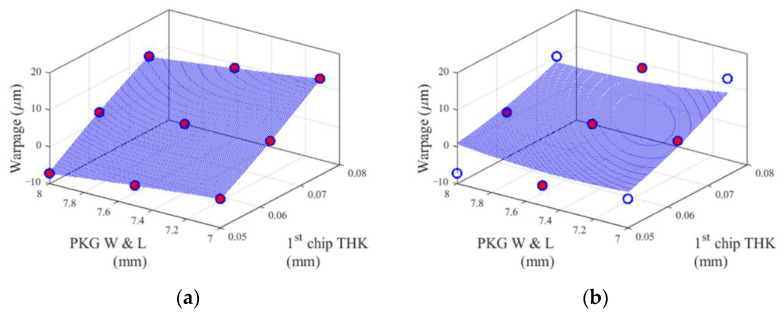
2D performance functions of design variables of (**a**) Bivariate Cut HDMR and (**b**) CCD, where design variables other than package width and length and 1st chip thickness are kept at their mean values.

Figure 9 and Figure 10 show the two predicted surfaces (cut-planes), which were studied in the TFBGA application. Each figure has the identical nine dots (warpage values obtained from the FE model) in (a) and (b). Red dots are the sample points used to construct for each metamodel. Blank dots are the sample points that were used to construct the Bivariate Cut-HDMR metamodel but not used to construct the CCD metamodel.

The surfaces (cut-planes) in Figure 9a and Figure 10a were constructed by Bivariate Cut-HDMR (quadratic spline interpolation) with two sets of nine sample points shown in the figures. There is no error between warpage values obtained from FE (dots) modeling and the predicted surfaces.

The surfaces in Figure 9b and Figure 10b were plotted by the CCD metamodel obtained from 1045 sample points. The five sample points shown in Figure 9b and Figure 10b were just a small portion of the total 1045 sample points used to construct the CCD metamodel (a second-order polynomial function). This attempt for CCD to fit all 1045 sample points inevitably produces the discrepancy between true warpage values (dots) and predicted surfaces in the entire design domain, especially in the corners, as shown in Figure 9b and Figure 10b.

#### 4.2.3. Prediction of Boundary-Planes

The example in Figure 8 (N=3) shows five sample points on the boundary-planes of both metamodels. It is important, however, to note that there are lesser or no sample points on the boundary-planes of both metamodels when the number of input variables (*N*) increases. On the boundary-planes of the TFBGA application (N=10), there were no sample point for Bivariate Cut-HDMR and only four sample points for CCD.

**Figure 11:**
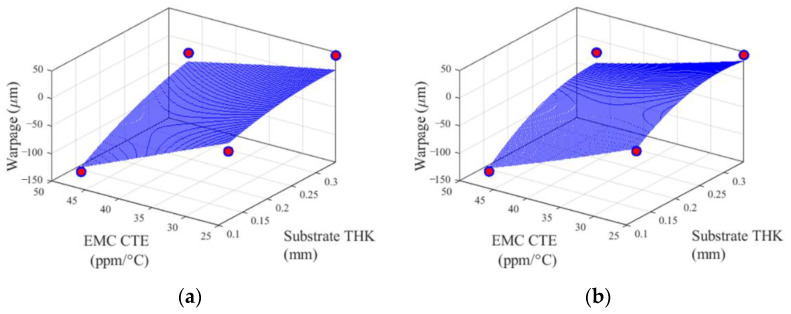
2D performance functions of (**a**) Bivariate Cut-HDMR and (**b**) CCD, where design variables other than EMC CTE and substrate thickness are kept at their maximum values.

Figure 11 shows two predicted surfaces (boundary-planes) of the TFBGA application. Variables other than the two variables shown in the plots were kept at their maximum values, i.e., it represents one of the boundary-planes in the design domain. Red dots in (b) are the sample points used to construct the CCD metamodel. They also appear in (a), although they are not used for Bivariate Cut-HDMR.

The Bivariate Cut-HDMR surface (boundary-planes) of Figure 11a are plotted by 201 sample points, while the CCD surface of Figure 11b are plotted by 1045 sample points including the four sample points on the boundary-plane. It is noteworthy that the predicted 2D performance function of Bivariate Cut-HDMR is similar to the CCD surface, despite the fact that CCD utilizes four sample points on the boundary-plane, but Bivariate Cut-HDMR does not.

## 5. Conclusions

Bivariate Cut-High Dimensional Model Representation (Bivariate Cut-HDMR) was implemented successfully for the warpage problem of a thin flat ball grid array package with 10 design variables. The implementation with three uniformly distributed sample points (*s* = 3) in conjunction with quadratic spline interpolation allowed for comparing its performance with a metamodel based on Central Composite Design (CCD).

The performance of both metamodels were evaluated by two well-known error metrics: *R*-squared and Relative Average Absolute Error (*RAAE*). The results were compared with the performance of CCD: the *R*-squared values of CCD and Cut-HDMR were 0.9662 and 0.9855, respectively; the *RAAE* values of CCD and Cut-HDMR were 0.1472 and 0.0880, respectively.

The outcome was remarkable. Bivariate Cut HDMR used only one-fifth of sample points (201 sample points) required by CCD (1045 sample points); however, Bivariate Cut-HDMR did not compromise the accuracy in both error metrics compared to CCD, which was confirmed by more direct and quantitative comparisons using the absolute errors of the Monte Carlo simulation (MCS) sample points.

Two technical reasons for the outstanding performance of Bivariate Cut-HDMR were discussed:

(1) Sampling scheme: the sample points of Bivariate Cut-HDMR were utilized to construct the first-order and second-order component functions, while the sample points of CCD were aimed to cover the boundaries of a design domain.
(2) Predictions of cut-planes and boundary-planes: Bivariate Cut-HDMR predicted cut-planes more accurately despite the smaller number of sample points, while both techniques produced similar accuracy for boundary-plane predictions.

## Figures and Tables

**Table 1:** Properties of materials used in the TFBGA package.

Material	Young’s Modulus (GPa)	Poisson’s Ratio	CTE (ppm/°C)		*T*_g_ (ׄ°C)
			A_1_ (<*T*_g_)	A_2_ (>*T*_g_)	
Silicon die	130	0.23	2.8		--
DAF	2.2 @ 25 °C	0.3	65.3	162.9	138
	0.98 @ 100 °C				
	0.008 @ 200 °C				
Substrate	17.5	0.3	15 (in-plane)		--
			61.5 (out-of-plane)		
EMC	29.237 @ 25 °C	0.21	9.12	36	137.5
	14.030 @ 125 °C				
	1.932 @ 175 °C				
	1.498 @ 235 °C				

**Table 2:** Dimensions of TFBGA package.

Structure	Length × Width × Thickness
1st Die (mm)	13 × 11 × 0.575
1st DAF (mm)	13 × 11 × 0.025
2nd Die (mm)	11 × 9 × 0.575
2nd DAF (mm)	11 × 9 × 0.025
Substrate (mm)	15 × 15 × 0.13
EMC (mm)	15 × 15 × 0.55

**Table 3:** Design variables of the TFBGA package.

Variable	Physical Meaning	Range of Design Space	Mean
*x*_1_	EMC thickness (mm)	0.25–0.85	0.55
*x*_2_	Substrate thickness (mm)	0.12–0.34	0.23
*x*_3_	1st chip thickness (mm)	0.050–0.075	0.0625
*x*_4_	2nd chip thickness (mm)	0.050–0.075	0.0625
*x*_5_	1st DAF thickness (mm)	0.02–0.025	0.0225
*x*_6_	2nd DAF thickness (mm)	0.01–0.02	0.015
*x*_7_	EMC CTE above *T*_g_ (ppm/°C)	25–47	36
*x*_8_	Substrate CTE (ppm/°C)	12–18	15
*x*_9_	Substrate modulus (GPa)	7.5–27.5	17.5
*x*_10_	Half of PKG width and length (mm)	7–8	7.5

**Table 4:** Number of sample points required for Central composite design (CCD) and Bivariate Cut HDMR with *s* = 3.

		Number of Sample Points		Ratio of Two Numbers of Sample Points RP
*N*	*s*	CCD $P=1+2N+2^{N}$	Bivariate Cut-HDMR $R=1+2N+\frac{N\left(N-1\right)}{2}{\left(2\right)}^{2}$	
2	3	9	9	100%
3	3	15	19	127%
4	3	25	33	132%
5	3	43	51	119%
6	3	77	73	95%
7	3	143	99	69%
8	3	273	129	47%
9	3	531	163	31%
10	3	1045	201	19%
11	3	2071	243	12%
12	3	4121	289	7%
13	3	8219	339	4%

**Table 5:** Performance metrics of Bivariate Cut-HDMR and CCD.

N	s	Method	Number of Sample Points	R-Squared	RAAE
10	3	Bivariate Cut-HDMR	201	0.9855	0.0880
		CCD	1045	0.9662	0.1472

## Data Availability

The data presented in this study are available in Supplementary Materials.

## Supplementary Materials

The following are available online at https://www.mdpi.com/article/10.3390/ma14164619/s1, Table S1: Bivariate Cut-HDMR sample points, Table S2: CCD sample points, Table S3: MCS sample points.
